# Mechanisms Underlying Activation of α_1_-Adrenergic Receptor-Induced Trafficking of AQP5 in Rat Parotid Acinar Cells under Isotonic or Hypotonic Conditions

**DOI:** 10.3390/ijms17071022

**Published:** 2016-06-28

**Authors:** Aneta M. Bragiel, Di Wang, Tomasz D. Pieczonka, Masayuki Shono, Yasuko Ishikawa

**Affiliations:** 1 Department of Medical Pharmacology, Institute of Biomedical Sciences, Tokushima University Graduate School, 3-18-15, Kuramoto-cho, Tokushima 770-8504, Japan; aneta.bragiel@gmail.com (A.M.B.); wangdic@126.com (D.W.); c301251017@tokushima-u.ac.jp (T.D.P.); 2 Support Center for Advanced Medical Sciences, Institute of Biomedical Sciences, Tokushima University Graduate School, 3-18-15, Kuramoto-cho, Tokushima 770-8504, Japan; masa@md.tokushima-u.ac.jp

**Keywords:** aquaporin-5, α_1A_-adrenoceptor, α_1B_-adrenoceptor, α_1D_-adrenoceptor, calcium, protein G kinase, hypotonicity

## Abstract

Defective cellular trafficking of aquaporin-5 (AQP5) to the apical plasma membrane (APM) in salivary glands is associated with the loss of salivary fluid secretion. To examine mechanisms of α_1_-adrenoceptor (AR)-induced trafficking of AQP5, immunoconfocal microscopy and Western blot analysis were used to analyze AQP5 localization in parotid tissues stimulated with phenylephrine under different osmolality. Phenylephrine-induced trafficking of AQP5 to the APM and lateral plasma membrane (LPM) was mediated via the α_1A_-AR subtype, but not the α_1B_- and α_1D_-AR subtypes. Phenylephrine-induced trafficking of AQP5 was inhibited by ODQ and KT5823, inhibitors of nitric oxide (NO)-stimulated guanylcyclase (GC) and protein kinase (PK) G, respectively, indicating the involvement of the NO/ soluble (c) GC/PKG signaling pathway. Under isotonic conditions, phenylephrine-induced trafficking was inhibited by La^3+^, implying the participation of store-operated Ca^2+^ channel. Under hypotonic conditions, phenylephrine-induced trafficking of AQP5 to the APM was higher than that under isotonic conditions. Under non-stimulated conditions, hypotonicity-induced trafficking of AQP5 to the APM was inhibited by ruthenium red and La^3+^, suggesting the involvement of extracellular Ca^2+^ entry. Thus, α_1A_-AR activation induced the trafficking of AQP5 to the APM and LPM via the Ca^2+^/ cyclic guanosine monophosphate (cGMP)/PKG signaling pathway, which is associated with store-operated Ca^2+^ entry.

## 1. Introduction

Salivary secretion is regulated by both sympathetic and parasympathetic nerves [1]. Activation of M_1_- and M_3_-muscarinic acetylcholine receptors (mAChRs) and α_1_-adrenoceptors (ARs) induce an increase in the cytosolic concentration of calcium ([Ca^2+^]i) and stimulate salivary fluid secretion [2]. Activation of β-adrenoceptors activates protein kinase (PK) A and also induces secretion of saliva containing high concentrations of protein [1]. Aquaporins (AQPs) are integral membrane proteins that facilitate water transport across the cellular membranes [3]. Thirteen AQPs have been identified in mammals [4]. AQP5 is expressed highly in salivary glands, lacrimal glands, lung, and so on [5]. Studies with knockout mice lacking AQP5 show defective fluid secretion, indicating a pivotal role for AQP5 in salivary fluid secretion [6].

Saliva is secreted from three major (parotid, submandibular and sublingual) glands and numerous minor glands. The submandibular, sublingual and minor salivary glands continuously secrete saliva, whereas the parotid glands do not contribute to unstimulated salivary secretion, but contribute to stimulated secretion [1,7]. In in vitro studies using rat parotid gland slices, the interactions of acetylcholine (ACh) and epinephrine with M_3_-mAChRs [8] and α_1_-ARs [9], respectively, induce a rapid trafficking of AQP5 to the apical plasma membrane (APM) via the enhanced [Ca^2+^]i. In vivo studies using confocal microscopy demonstrated that activation of M_3_- and M_1_-mAChRs induces the rapid trafficking of AQP5 associated with lipid rafts from the cytoplasm to the APM [10,11,12]. The activation of M_3_- and M_1_-mAChRs also induces the release of AQP5 and lipid rafts into the saliva [13]. AQP5 is identified in parotid gland exosomes [14], which are packed in cytosolic multivesicular bodies. Upon fusion of multivesicular bodies with the APM in a Ca^2+^-triggered manner, AQP5 is released into the saliva.

The α_1_-ARs subfamily consists of three (α_1A_, α_1B_ and α_1D_) subtypes [15]. In rat parotid glands, the presence of mRNA and receptor protein for both the α_1A_- and α_1B_-AR subtypes, along with detectable α_1D_-AR mRNA, has been confirmed [16,17]. α_1_-AR antagonists are widely used in the treatment of lower urinary tract symptoms (LUTS) [18,19,20,21,22] and cardiovascular diseases [23]. However, α_1_-AR antagonists cause dry mouth as a side effect [24]. The α_1A_-AR is linked to G-protein G_q/11_ signaling and a subsequent rise in [Ca^2+^]i, while the α_1B_-AR activates the mitogen-activated kinase (MAPK)/extracellular signal-regulated kinase 1 and 2 (ERK1/2) [25,26].

In the present work, we investigated the mechanisms underlying the translocation of AQP5 in association with the α_1_-AR, as well as the associated signal transduction. Furthermore, we investigated the responsiveness of AQP5 trafficking to an α_1_-AR agonist under different osmolality, in light of the recent reports that hyper- and hypo-tonicity induce trafficking of AQP5 in several tissues [27,28,29,30,31]. The hypotonic conditions activate the transient receptor potential (TRPV) 4 and induce the trafficking of AQP5 to the APM in human salivary gland cells [28] and reduction of AQP5 in lung epithelial cells [29]. In the cochlea, the hypertonicity induces the trafficking of AQP5 to the APM [30]. On the other hand, it is reported that TRPV4 seems to play a role during cholinergic activation in immortalized salivary cell line [32]. To clarify the relationship between α_1_-AR activation, osmolality and TRPV4, the mechanisms underlying α_1_-AR induced the trafficking of AQP5 were investigated under different osmolality in rat parotid glands. 

## 2. Results

### 2.1. α_1_-AR Agonist-Induced Trafficking of AQP5 and Ganglioside GM1 to the APM and Lateral Plasma Membrane (LPM)

**Figure 1:**
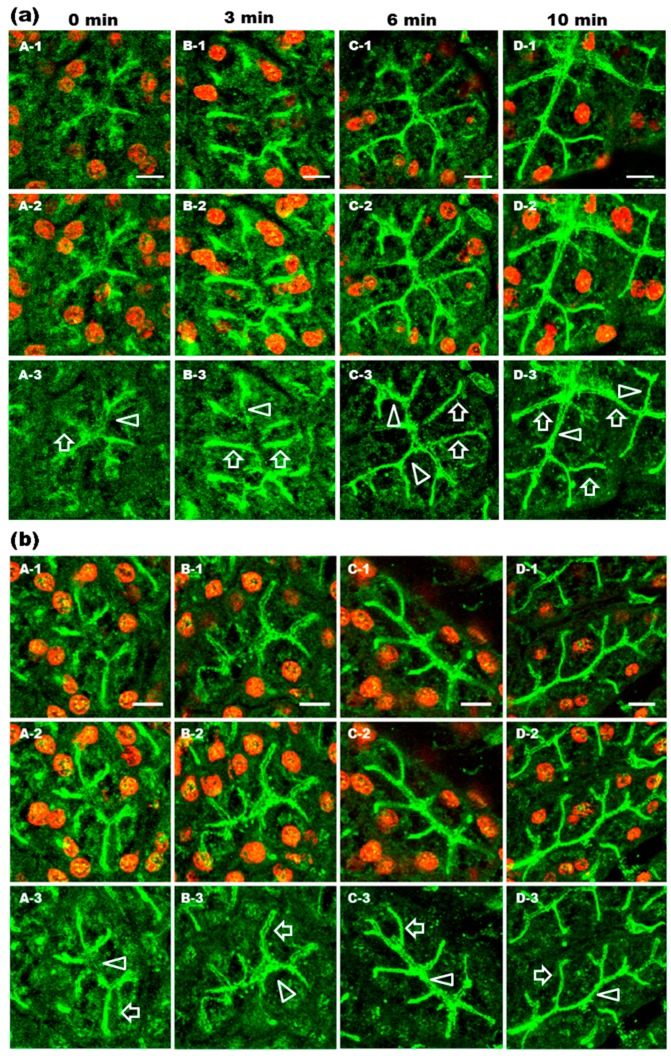
Immunohistochemical analysis of aquaporin-5 (AQP5) and ganglioside GM1 distributions in rat parotid acinar cells after phenylephrine injection. Parotid glands were obtained from rats 0 (**A**), 3 (**B**), 6 (**C**), and 10 (**D**) min after phenylephrine (0.25 mg/kg) injection. Tissue sections were fixed in ethanol followed by incubation with anti-AQP5 (**a**) and anti-GM1 (**b**) antibodies. Alexa-488 was used to visualize AQP5 and GM1. Propidium iodide (PI) was used to stain nuclei. One section is presented in each single image (−1). Sixteen consecutive images produced by a confocal microscope were projected to generate a single image (−2). To get clear visualization of AQP5 localization in acinar cells, the 568 nm laser was turned off (−3). Arrow heads and arrows indicate apical plasma membrane (APM) and lateral plasma membrane (LPM), respectively. Scale bars: 10 µm.

Our previous studies demonstrated that AQP5 is translocated to the APM in response to activation of M_3_-mAChR [8] or α_1_-ARs [9] in vitro. Recently, published results from in vivo experiments revealed that the activation of M_3_- and M_1_-mAChRs induces the translocation of AQP5, together with lipid rafts, from the cytoplasm to the APM [11,12]. In the present study, we used confocal microscopy to directly visualize the cellular distribution and translocation of AQP5 in rat parotid glands under α_1_-AR activation. Phenylephrine (nonspecific α_1_-AR agonist) (0.25 mg/kg) was injected into the tail vein of rats. After 0 min (control), AQP5 (Figure 1a; A) and GM1 (Figure 1b; A) labeling was present in the APM, scattered in LPM, and large amounts of AQP5 and GM1 were diffusely distributed throughout the cytoplasm and in the apical plasmalemmal region. GM1 was used as a marker of lipid rafts [33]. After 3 min, AQP5 (Figure 1a; B) and GM1 (Figure 1b; B) showed similar staining patterns to those of control (Figure 1a,b; A). However, after 6 to 10 min, AQP5 (Figure 1a; C and D) and GM1 (Figure 1b; C and D) were located predominantly in the APM and LPM.

### 2.2. Prevention of α_1_-AR Agonist-Induced Trafficking of AQP5 and GM1 by Phentolamine

**Figure 2:**
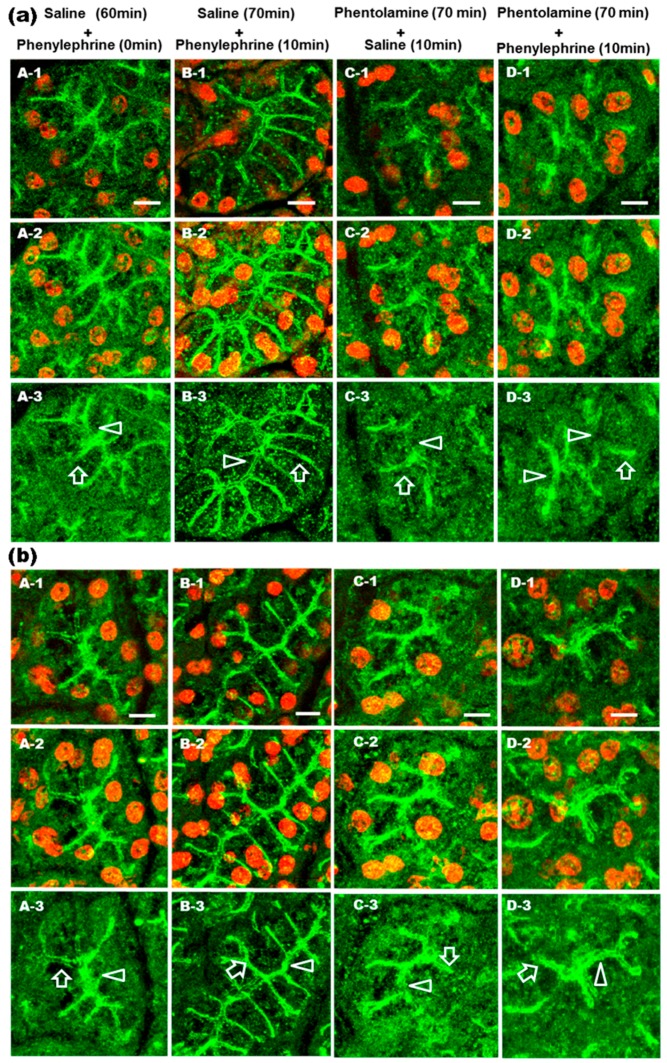
Immunohistochemical analysis of AQP5 and GM1 distributions in rat parotid acinar cells after phenylephrine and phentolamine injection. Rats were intraperitoneally injected with phentolamine (10 mg/kg) (**C** and **D**) or saline (**A** and **B**). After 60 min, phenylephrine (0.25 mg/kg) (**B** and **D**) was intravenously injected. Parotid glands were obtained from rats 0 (**A**) and 10 (**B**–**D**) min after phenylephrine or saline injection. Alexa-488 was used to visualize AQP5 (**a**) and GM1 (**b**). PI was used to stain nuclei. One section is presented in each single image (−1). Sixteen consecutive images produced by a confocal microscope were projected to generate a single image (−2). To get clear visualization of AQP5 localization in the acinar cells, the 568 nm laser was turned off (−3). Arrow heads and arrows indicate APM and LPM, respectively. Scale bars: 10 µm.

Previous in vitro experiments showed that phentolamine (nonspecific α_1_-AR antagonist) inhibited the epinephrine-induced increases of AQP5 in the APM [9]. To visualize the effect of an α_1_-AR antagonist on the subcellular distribution of AQP5 (Figure 2a) and GM1 (Figure 2b) in vivo, phentolamine or saline were injected intraperitoneally. Sixty minutes after saline-injection, AQP5 (Figure 2a; A) and GM_1_ (Figure 2b; A) staining were observed throughout the cytoplasm, with little AQP5 in the APM and LPM. Sixty minutes after the phentolamine or saline injection, phenylephrine (0.25 mg/kg) was injected into the tail vein (Figure 2a,b; B and D). Ten minutes later, AQP5 (Figure 2a; D) and GM_1_ (Figure 2b; D) were not accumulated in the APM and LPM of phentolamine-injected rat compared with non-phentolamine-injected rats (Figure 2a,b; B). Saline was injected into the tail vein 60 min after phentolamine-injection (Figure 2a,b; C). Ten minutes later, AQP5 (Figure 2a; C) and GM1 (Figure 2b; C) showed similar staining patterns to those of control (Figure 2a,b; A). These results indicate that phentolamine prevented the phenylephrine-induced increases in AQP5 and GM1 in the APM and LPM.

### 2.3. Purity of APM Isolated from Rat Parotid Gland

To evaluate the purity and cross-contamination of the APM and basolateral plasma membrane (BLM), isolated fractions were examined for the presence of enzymatic activities of γ-glutamyl transpeptidase (γ-GT) and K^+^-stimulated *p*-nitrophenyl phosphatase (K^+^-NPPase) markers of APM and BLM, respectively. The γ-GT activities in APMs and BLMs were 24 and 4 mU/mg protein, respectively (Table S1). The K^+^-NPPase activities were 20 and 109 mU/mg protein in APMs and BLMs, respectively (Table S1). When the γ-GT activity in APM was more than five times higher than in BLM, and the K^+^-NPPase activity was less than one-fifth of that in BLM, APM samples were used in further experiments. 

### 2.4. Effects of Antagonists of α_1_-AR Subtypes on the Phenylephrine-Induced Trafficking of AQP5

**Figure 3:**
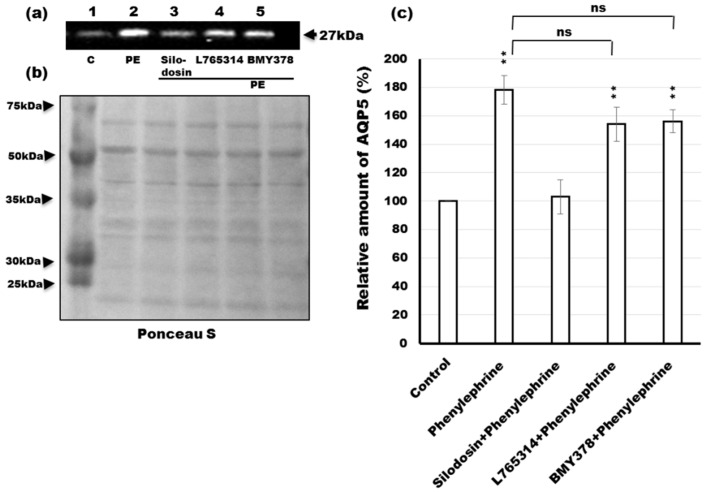
Effects of α_1_-AR subtype antagonists on phenylephrine-induced increases in AQP5 levels in the APM. (**a**) Tissue slices from rat parotid glands were incubated for 10 min at 37 °C without (lane 1) or with 1 µM phenylephrine (PE) (lanes 2–5) plus 10 µM silodosin (lane 3), 10 µM L765314 (lane 4) and 10 µM BMY7378 (lane 5). The 5 µg of APM fraction protein was then loaded on sodium dodecyl sulfate polyacrylamide gel electrophoresis (SDS-PAGE) and processed by immunoblot analysis with anti-AQP5 antibody; (**b**) The blotted membrane was stained by Ponceau S; (**c**) Densitometric analysis was carried out normalizing to total protein amount by staining nitrocellulose membrane with Ponceau S solution. The amount of AQP5 in the APM, measured by the intensity of chemiluminescence, was shown as a percentage of the value for control tissue. Values are expressed as mean ± SE of three independent experiments. ** *p* < 0.01 vs. the value for control tissue. ns: not significant.

Some studies describe a high incidence of dry mouth as a side effect of treatment with α_1_-AR antagonists [24]. Therefore, to identify the specific α_1_-AR subtype accountable for the phenylephrine-induced trafficking of AQP5, rat parotid tissue slices were incubated with specific α_1_-AR antagonists. Incubation of tissues for 10 min with 1 µM phenylephrine induced a 1.8-fold increase in the amount of AQP5 in the APM (Figure 3a,c; lane 2). The effect of phenylephrine was inhibited by the α_1A_-adrenergic specific antagonist silodosin (10 µM) (Figure 3a,c; lane 3). However, neither the α_1B_-AR antagonist L765314 (10 µM) (Figure 3a,c; lane 4) nor the α_1D_-AR antagonist BMY7378 (10 µM) (Figure 3a,c; lane 5) had significant effects on the amount of AQP5 in the APM. These results suggest that phenylephrine acts at the α_1A_-AR to promote the trafficking of AQP5 to the APM in rat parotid cells.

In the western blotting, Ponceau S staining of nitrocellulose membrane was used to assess equal loading of proteins (Figure 3b). The levels of protein were corrected for whole protein loading [34] and Ponceau S staining is considered to be a valuable tool for normalization of western blots due to its advantages over housekeeping proteins [35].

### 2.5. Prevention of Phenylephrine-Induced Trafficking of AQP5 by α_1A_-AR Antagonist

**Figure 4:**
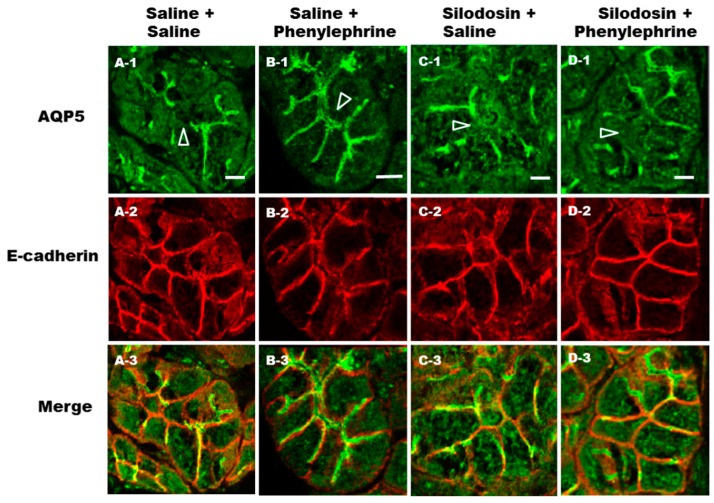
Immunohistochemical analysis of AQP5 and *E*-cadherin distributions in rat parotid acinar cells after phenylephrine and silodosin administration. Rats were treated with silodosin (a daily dose of 1 mg/kg p.o.) (**C** and **D**) or saline (**A** and **B**) once daily for 1 week. Subsequently, phenylephrine (0.25 mg/kg) (**B** and **D**) or saline (**A** and **C**) was intravenously injected and parotid glands were subjected to immunohistochemical analysis with anti-AQP5 and anti-*E*-cadherin antibodies. Secondary antibodies coupled to Alexa-488 or 568 were used to visualize AQP5 (**A-1** to **D-1**) and *E*-cadherin (**A-2** to **D-2**), respectively. Arrow heads indicate APM. Scale bars: 10 µm.

In light of the results obtained from the in vitro experiment, we next sought to determine if AQP5 trafficking in vivo was disrupted by the α_1A_-AR specific antagonist silodosin. In order to directly visualize the effect of silodosin on phenylephrine-induced translocation of AQP5 to the APM and LPM, phenylephrine (0.25 mg/kg) was injected after the oral administration of silodosin at a daily dose of 1 mg/kg for 1 week. In the parotid glands of control rats, AQP5 labeling (Figure 4; A-1) was present in the APM, scattered in LPM, throughout the cytoplasm and in the apical plasmalemmal region. After 10 min of phenylephrine injection, AQP5 was mainly localized in the APM and LPM (Figure 4; B-1). Silodosin plus saline (Figure 4; C-1) and silodosin plus phenylephrine (Figure 4; D-1) treatment of rat parotid tissue resulted in inhibition of AQP5 trafficking to the APM and LPM. Confocal laser microscopy demonstrated that the AQP5 staining was confined to the same compartments as in control rats. *E*-cadherin is considered to be a marker of the LPM [36]. To confirm trafficking of AQP5 to the LPM, an antibody against *E*-cadherin was used. *E*-cadherin immunolocalization was similar among the saline (Figure 4; A-2), saline plus phenylephrine (Figure 4; B-2), silodosin plus saline (Figure 4; C-2) and silodosin plus phenylephrine (Figure 4; D-2) treated rat parotid acinar cells. The results confirm that phenylephrine-induced AQP5 is trafficking to the APM and LPM via α_1A_-AR.

### 2.6. Effects of ODQ and KT5823 on AQP5 Levels in the APM of Phenylephrine-Stimulated Parotid Tissue

**Figure 5:**
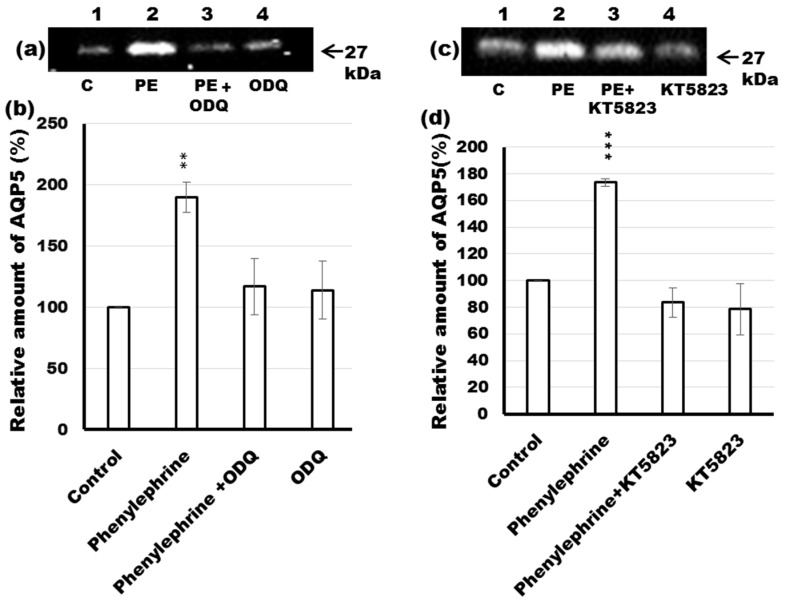
Effects of the antagonists ODQ and KT5823 on phenylephrine-induced increases in AQP5 levels in the APM. (**a**) Tissue slices from rat parotid glands were incubated for 10 min at 37 °C in the absence (lanes 1 and 4) or presence of 1 µM phenylephrine (lanes 2 and 3) plus 10 µM ODQ (lanes 3 and 4). The 5 µg of APM fraction protein was loaded on SDS-PAGE and processed by immunoblot analysis with anti-AQP5 antibody. Densitometric analysis was carried out normalizing to total protein amount by staining membrane with Ponceau S solution. The membrane stained with Ponceau S was shown in Figure S1; (**b**) The amount of AQP5 in the APM, measured by the intensity of chemiluminescence, was shown as a percentage of the value for control tissue; (**c**) Tissue slices from rat parotid glands were incubated for 10 min at 37 °C in the absence (lanes 1 and 4) or presence of 1 µM phenylephrine (lanes 2 and 3) plus and 10 µM KT5823 (lanes 3 and 4). The 5 µg of APM fraction protein was loaded on SDS-PAGE and processed by immunoblot analysis with anti-AQP5 antibody; (**d**) Densitometric analysis was carried out normalizing to total protein amount by staining membrane with Ponceau S solution. The amount of AQP5 in the APM, measured by the intensity of chemiluminescence, was shown as a percentage of the value for control tissue. Values are expressed as mean ± SE of three independent experiments. ** *p* < 0.01, *** *p* < 0.01 vs. the value for control tissue.

In a previous study, the nitric-oxide (NO)/cGMP signaling pathway has been shown to play a pivotal role in Ca^2+^ homeostasis in the mAChR-stimulated rise in AQP5 levels in the APM of rat parotid tissue [37]. To investigate whether the NO/cGMP/PKG pathway affects AQP5 levels in the APM of phenylephrine-stimulated parotid gland, the tissue was incubated with ODQ or KT5823, inhibitors of NO-stimulated guanylyl cyclase (GC) and PKG, respectively. Treatment of the tissue for 10 min with 10 µM of KT5823 prevented the increase in AQP5 levels in the APM induced by phenylephrine (Figure 5c,d; lane 3). Similar results were obtained after incubation of the gland with 10 µM ODQ, in which the inhibitor abrogated the phenylephrine-induced increase of AQP5 in the APM (Figure 5a,b; lane 3). In both cases, treatment of the gland with the inhibitors alone did not have a significant effect on the level of AQP5 in the APM (Figure 5a–d; lane 4). The present results support the participation of the NO/cGMP/PKG signaling pathway in the α_1_-adrenoceptor-stimulated rise in AQP5 trafficking to the APM of the rat parotid gland.

### 2.7. Effect of Differential Osmolality on AQP5 Trafficking to the APM in Parotid Tissues

**Figure 6:**
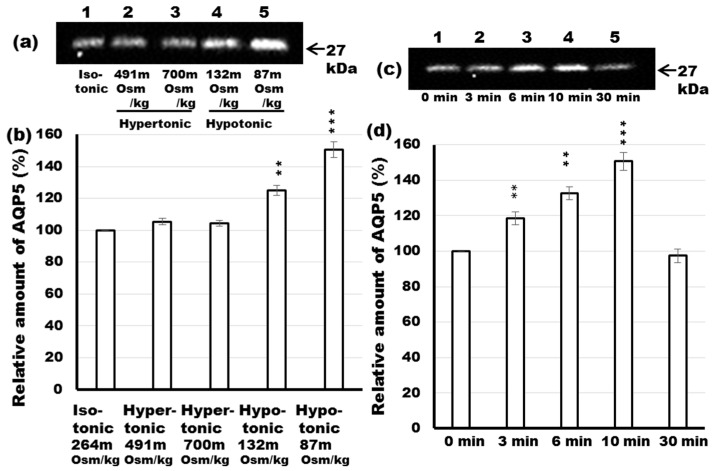
Effect of hypotonicity or hypertonicity on the translocation of AQP5 in rat parotid glands. (**a**) Tissue slices from rat parotid glands were incubated for 10 min at 37 °C in isotonic (lane 1), hypertonic (lanes 2 and 3) and hypotonic (lanes 4 and 5) solutions. Hypertonic and hypotonic solutions were made by addition of higher tonicity solution and by dilution with water, respectively. The 5 µg of APM fraction protein was loaded on SDS-PAGE and processed by immunoblot analysis with anti-AQP5 antibody; (**b**) Densitometric analysis was carried out normalizing to total protein amount by staining membrane with Ponceau S solution and values were expressed as a percentage of the control. The membrane stained with Ponceau S was shown in Figure S1. Values are expressed as mean ± SE of three to six independent experiments; (**c**) Parotid tissue was incubated for 0, 3, 6, 10, and 30 min in hypotonic solution (87 mOsm/kg) (lanes 1–5). At the designated times, the tissue was homogenized, the APM was isolated and 5 µg of sample was subjected to immunoblot analysis with anti-AQP5 antibody; (**d**) Densitometric analysis was carried out normalizing to total protein amount by staining membrane with Ponceau S solution and values were shown as a percentage of the control. The membrane stained with Ponceau S was shown in Figure S1. Values are expressed as mean ± SE of three independent experiments. ** *p* < 0.01, *** *p* < 0.001 vs. control (lane 1).

Hypotonicity induced the trafficking of AQP5 to the APM in cultured cells from human submandibular and parotid glands [28], whereas it reduced AQP5 abundance in lung epithelial cells [29]. Hyperosmolar perfusion of the perilymphatic fluid induced a significant increase of AQP5 in the APM, but decreased AQP5 in the cytoplasm, in cochlea [30]. To evaluate the physiological relevance of changes in tonicity-triggered AQP5 translocation, rat parotid tissue slices were incubated in isotonic (264 mOsm/kg), hypertonic (491 and 700 mOsm/kg) and hypotonic (132 and 87 mOsm/kg) solutions, and at the designated time, APM fractions were prepared and submitted to immunoblot analysis. Results revealed that AQP5 protein was induced maximally (1.6-fold) when the tissue was incubated in 87 mOsm/kg solution (Figure 6a,b; lane 5) and to a lesser extent (1.25-fold) by incubation in 132 mOsm/kg solution (Figure 6a,b; lane 4). Results also showed that AQP5 surface localization did not change significantly after 10 min of hypertonic challenges, neither at 491 nor at 700 mOsm/kg (Figure 6a,b; lanes 2 and 3). These data suggest that hypoosmolarity and its threshold, but not hyperosmolarity, induce AQP5 translocation to the APM in the rat parotid gland.

In our in vivo experiments using the rat parotid gland, α_1_-adrenoceptor agonist-induced trafficking of AQP5 showed a time-dependent increase in the APM, with a peak at 6–10 min. To examine whether hypotonicity-induced increases of AQP5 in the APM are also time-dependent, parotid sections were analyzed at 0, 3, 6, 10 and 30 min after incubation in hypotonic solution. For the control, parotid tissue was incubated for 0 min in hypotonic solution (Figure 6c,d; lane 1). AQP5 protein increased in the APM at 3 min (Figure 6c,d; lane 2) after exposure to hypotonic solution and peaked at 6–10 min, reaching 130% and 150% of the control (0 min) level, respectively (Figure 6c,d; lanes 3 and 4). Prolonged incubation (30 min) in hypotonic solution resulted in a reduction of AQP5 to the baseline level (Figure 6c,d; lane 5). These data are highly similar to the time-dependent increase of AQP5 levels in the APM of rat parotid tissues induced by phenylephrine.

**Figure 7:**
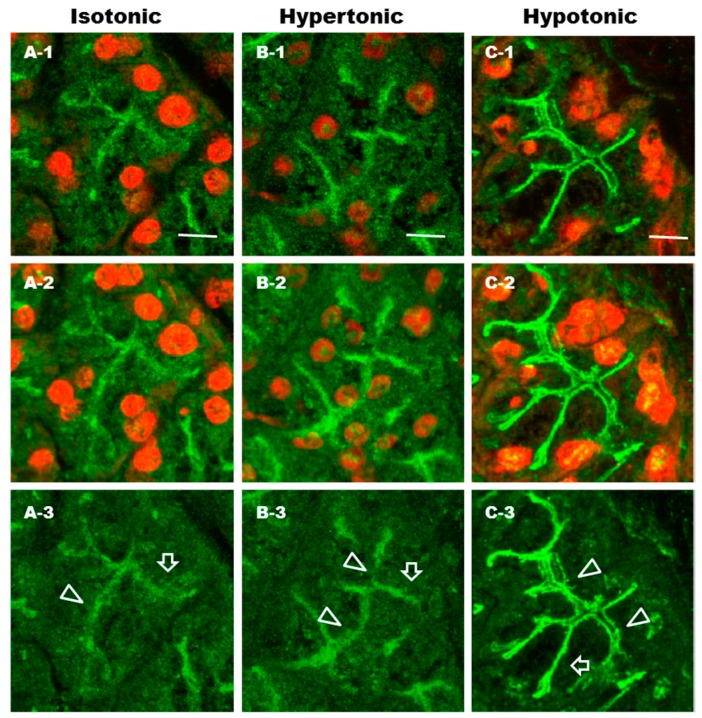
Immunohistochemical analysis of AQP5 distribution in rat parotid acinar cells under isotonic, hypertonic and hypotonic conditions. Parotid gland tissue sections were incubated for 10 min at 37 °C in isotonic (**A**), hypertonic (**B**) and hypotonic (**C**) solutions. Tissue sections were fixed in ethanol followed by incubation with anti-AQP5 antibody. Secondary antibody coupled to Alexa-488 was used to visualize AQP5. PI was used to stain nuclei. One section is presented in each single image (−1). Sixteen consecutive images produced by a confocal microscope were projected to generate a single image (−2). To get clear visualization of the AQP5 localization in acinar cells, the 568 nm laser was turned off (−3). Arrow heads and arrows indicate APM and LPM, respectively. Scale bars: 10 µm.

To visualize hypotonicity-induced trafficking of AQP5, parotid sections were incubated for 10 min in isotonic (Figure 7; A), hypertonic (Figure 7; B) and hypotonic (Figure 7; C) solutions and then were analyzed by confocal microscopy. In isotonic and hypertonic conditions, large amount of AQP5 were diffusely distributed throughout the cytoplasm, and the apical plasmalemmal region and scattered in LPM. In hypotonic condition, AQP5 was distributed predominantly in the APM and LPM. Thus, hypotonicity induced the trafficking of AQP5 to the APM and LPM in rat parotid glands.

### 2.8. Effects of Calcium Channel Blockers on Phenylephrine- and Hypotonicity-Induced Translocation of AQP5 in Rat Parotid Tissue

**Figure 8:**
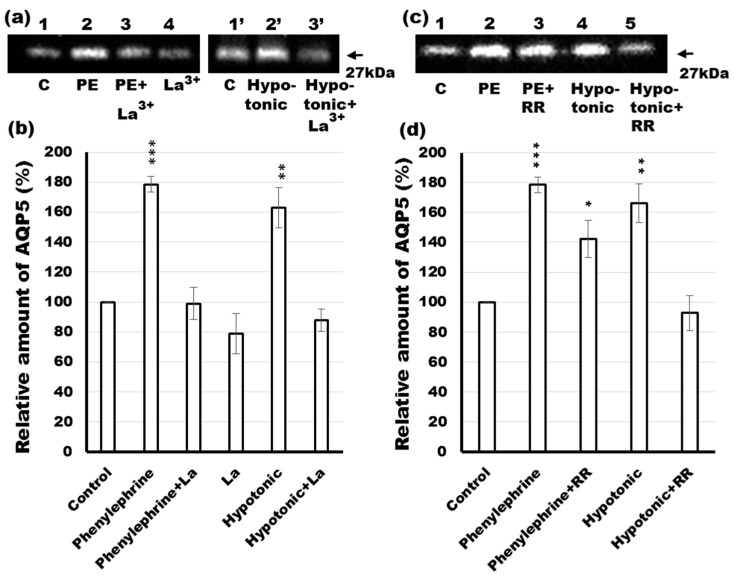
Effects of lanthanum chloride (La^3+^) and ruthenium red (RR) on phenylephrine- or hypotonicity-induced increases in AQP5 levels in the APM. (**a**) Tissue slices from rat parotid glands were incubated for 10 min at 37 °C in isotonic (lanes 1-4 and 1’) or hypotonic (lanes 2’ and 3’) solution without (lanes 1 and 4) or with 1 µM phenylephrine (lanes 2 and 3) plus 3 mM La^3+^ (lanes 3, 3’ and 4). The 5 µg of APM fraction protein was prepared and processed by immunoblot analysis with anti-AQP5 antibody; (**b**) Densitometric analysis was carried out normalizing to total protein amount by staining membrane with Ponceau S solution and values were expressed as a percentage of the control. The membrane stained with Ponceau S was shown in Figure S1; (**c**) Tissue slices from the glands were incubated for 10 min at 37 °C in control (lane 1), phenylephrine (lane 2), phenylephrine with RR (lane 3), hypotonic (lane 4) and hypotonic with phenylephrine solutions (lane 4). The osmolality of the solutions were 261 mOsm/kg (lanes 1–3) and 87 mOsm/kg (lanes 4 and 5) with 1 µM of phenylephrine and 10 µM of RR as the final concentration. The 5 µg of APM fraction protein was loaded on SDS-PAGE and processed by immunoblot analysis with anti-AQP5 antibody; (**d**) Densitometric analysis was carried out normalizing to total protein amount by staining membrane with Ponceau S solution and values were expressed as a percentage of the control. Values are expressed as mean ± SE of three to six independent experiments. * *p* < 0.05, ** *p* < 0.01, *** *p* < 0.001 vs. control.

Elevation of [Ca^2+^]i occurs by at least two routes in salivary glands. One is the release from endoplasmic reticulum Ca^2+^ stores to the cytoplasm [2], while the other involves direct Ca^2+^ entry to the cytoplasm from the extracellular space [28]. The store-operated Ca^2+^ entry mechanism (SOCE) consists of calcium release-activated calcium channel protein (Orai) 1, transient receptor potential channel (TRPC) 1, TRPC3, and stromal interaction molecule (STIM) 1 as its critical components [2,38], and is activated by Ca^2+^ store depletion from endoplasmic reticulum. Ca^2+^ entry into stores is needed to sustain the elevation of [Ca^2+^]i and saliva secretion. In this experiment, we evaluated the effect of a SOCE and TRPV4 channel blocker on phenylephrine- and hypotonicity-induced translocation of AQP5 to the APM. Rat parotid gland slices were incubated with (Figure 8a,b; lanes 3, 3’ and 4) or without (Figure 8a,b; lanes 1, 2, 1’ and 2’) lanthanum chloride heptahydrate (La^3+^) (store-operated Ca^2+^ entry and TRPV antagonist) [39,40,41] in the presence (Figure 8a,b; lanes 2, 3) or absence (Figure 8a,b; lanes 1, 4) of phenylephrine and in isotonic (Figure 8a,b; lane 1’) or hypotonic (Figure 8a,b; lane 2’) solution. After 10 min of incubation, samples were subjected to further analysis. The results showed that 3 mM La^3+^ significantly decreased the amount of AQP5 in the APM of rat parotid acinar cells stimulated by phenylephrine (Figure 8a,b; lane 3) and hypotonicity (Figure 8a,b; lane 3’). These data suggest that SOCE and TRPV4-mediated extracellular influx of Ca^2+^ plays an important role in phenylephrine- and hypotonicity-induced trafficking of AQP5 in rat parotid glands, respectively.

It has been shown that transient receptor potential (TRPV) 4 mediates the influx of extracellular Ca^2+^ [42], and that AQP5 is necessary for the hypotonicity-induced activation of TRPV4 and for regulation of cell volume recovery [28]. In the present work, we examined the effects of inhibition pathways, connected with Ca^2+^, implicated in phenylephrine- and hypotonic-induced trafficking of AQP5 to the APM. We therefore incubated rat parotid tissue sections in isotonic (Figure 8c,d; lane 1), isotonic with phenylephrine (Figure 8c,d; lane 2), isotonic with phenylephrine and ruthenium red (RR) solutions (an inhibitor of multiple TRPV channels) [28,29] (Figure 8c,d; lane 3) as well as in hypotonic (Figure 8c,d; lane 4) and hypotonic with RR solutions (Figure 8c,d; lane 5) for 10 min and subjected the samples to western blotting. The obtained data showed that 10 µM RR, completely blocked the translocation of AQP5 to APM in response to hypotonicity (Figure 8c,d; lane 5 vs. 4). In contrast, the same concentration of RR did not significantly attenuate the phenylephrine-induced increase of AQP5 in APM (Figure 8c,d; lane 3 vs. 2). This phenomenon may be a consequence of inhibition of TRPV4 and a subsequent decrease in the intracellular Ca^2+^ concentration, which is known to be critical in AQP5 trafficking and fluid secretion in salivary glands. The above results suggest that although hypotonicity and phenylephrine are efficient stimulators of AQP5 trafficking, they may induce trafficking of the protein to APM via distinct mechanisms.

### 2.9. Phenylephrine-Induced AQP5 Translocation to the APM in Rat Parotid Tissues under Hypotonicity

**Figure 9:**
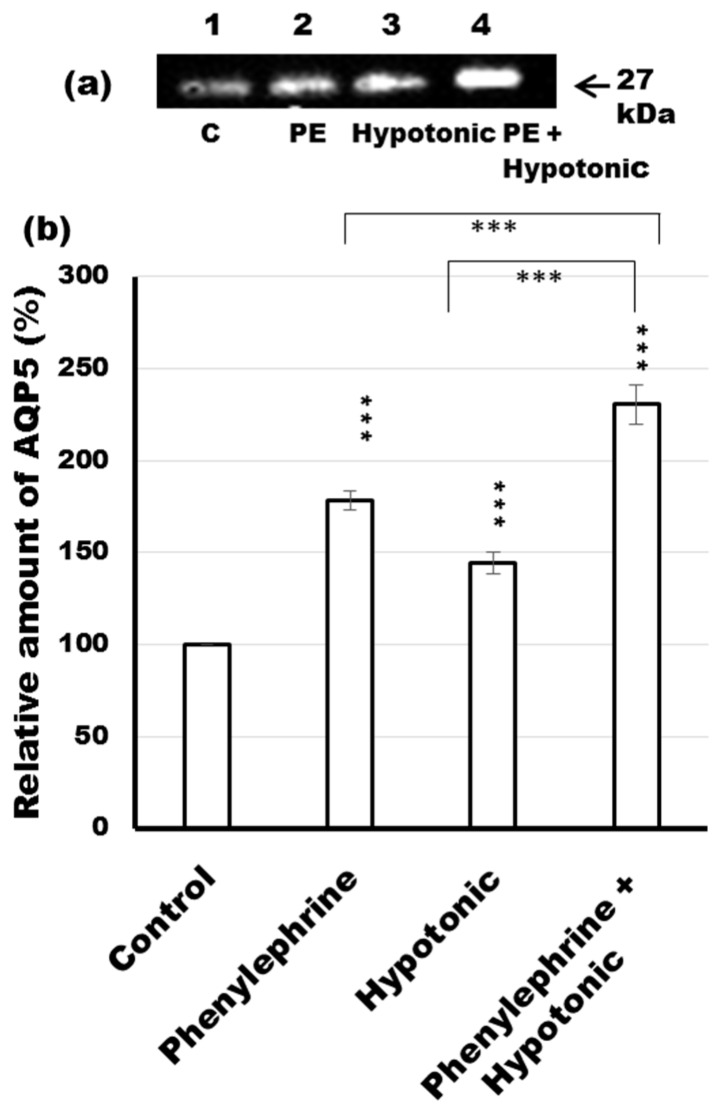
Phenylephrine-induced translocation of AQP5 in rat parotid tissues under hypotonicity. (**a**) Tissue slices from rat parotid glands were incubated for 10 min at 37 °C in control (lane 1), phenylephrine (lane 2), hypotonic (lane 3) and hypotonic with phenylephrine (lane 4) solutions. The osmolality of the solutions were 261 mOsm/kg (lanes 1, 2) and 87 mOsm/kg (lanes 3, 4) with 1 µM of phenylephrine as a final concentration. The 5 µg of APM fraction protein was loaded on SDS-PAGE and processed by immunoblot analysis with anti-AQP5 antibody; (**b**) Densitometric analysis was carried out normalizing to total protein amount by staining membrane with Ponceau S solution and values were expressed as a percentage of the control. The membrane stained with Ponceau S was shown in Figure S1. Values are expressed as mean ± SE of three independent experiments. *** *p* < 0.001 vs. control.

In order to investigate the combined effect of hypotonicity and phenylephrine-induced trafficking to the APM, parotid tissue slices were incubated with (Figure 9a,b; lanes 2 and 4) or without (Figure 9a,b; lanes 1 and 3) phenylephrine in isotonic solution (264 mOsm/kg) (Figure 9a,b; lanes 1 and 2) or hypotonic solution (87 mOsm/kg) (Figure 9a,b; lanes 3 and 4) for 10 min. Under isotonic condition, phenylephrine induced a 1.8-fold increase in AQP5 in the APM compared to the control (Figure 9, lane 2 vs. 1), while the incubation of tissue in hypotonic solution containing phenylephrine induced 1.7-fold greater increase in AQP5 than hypotonicity alone (Figure 9, lane 4 vs. 3). These data indicate that the extent of responsiveness of AQP5 trafficking to an α_1_-AR agonist under hypotonic condition is the same as that under isotonic condition.

## 3. Discussion

We previously showed that epinephrine acting at α_1_-ARs induces a rise in AQP5 levels in the APM of rat parotid glands by increasing [Ca^2+^]i. This induced increase of AQP5 was abrogated by phentolamine in vitro [9]. In the present study, we directly visualized the subcellular localization of AQP5 and ganglioside GM1 in rat parotid acinar cells stimulated by phenylephrine. Under control conditions, AQP5 and GM1 were distributed in large amounts throughout the cytoplasm and apical plasmalemmal region of parotid acinar cells. However, phenylephrine administration activated α_1_-adrenoceptor-induced trafficking of AQP5 and GM1 to the APM and LPM, peaking from 6 to 10 min after injection. We investigated the effect of phentolamine on the subcellular distribution of AQP5 and GM1 in rat parotid tissue. To restrain the tonic innervation of adrenergic nerve, phentolamine was injected into abdominal cavity of rats. Sixty minutes after phentolamine-injection, phenylephrine or saline was injected intravenously into the tail vein. Ten minutes after phenylephrine injection, AQP5 and GM1 remained in the same compartments as in the saline-injected rats. These results indicate that phentolamine inhibited the phenylephrine-induced increase in AQP5 and GM1 in the APM and LPM of rat parotid glands. 

The luminal membrane represents only 7.5% of the total plasma membrane of the rat parotid acinar cells, which constitute a very small area compared to BLM [43]. In the unstimulated conditions, the portion of AQP5 localized in the APM sufficiently supports salivary secretion. However, upon stimulation, when large volumes of water fluxes are required, the translocation of AQP5 to APM and additionally to the LPM of parotid gland, effectively increase the appropriate membrane surface of the cells thus, compensate its small apical area and transport comparably large volumes of fluid. Moreover, the results of AQP5 knockout studies showed the decrease in both paracellular and transcellular water transport in salivary glands, indicating that functions of these two pathways are linked [44]. In light of these results, it can also be speculated that trafficking of AQP5 to LPM could be the mechanism that supports water transport and saliva secretion through paracellular pathway in salivary glands stimulated by activation of α_1_-AR.

Next, we investigated the α_1_-AR subtypes in relation to phenylephrine-induced trafficking of AQP5. Silodosin, but not L765314 and BMY378, inhibited phenylephrine-induced trafficking of AQP5, suggesting that α_1_-AR activation-induced trafficking of AQP5 is mediated by the α_1A_-AR. The α_1A_-AR is distributed in the genitourinary system and plays a pivotal role in contraction of the vas deferens and the neck of the bladder [45]. Antagonists of the α_1A_-AR are important in the treatment of LUTS due to bladder obstruction in patients with decreased bladder blood flow [18], with benign prostatic hyperplasia [19,20], or with urolithiasis [21] and urethra contraction [22]. The α_1B_-AR subtype is localized in the spleen and nucleus accumbens, where it is involved in the regulation of spleen contraction [45] and locomotion [46]. Chronic activation of the α_1B_-AR leads to a shorter lifespan caused by cancer and neurodegeneration [47]. The α_1D_-AR subtype is present in blood vessels and regulates arterial blood pressure via vasocontraction [48]. Antagonists of the α_1D_-AR are thought to be therapeutically important in cardiovascular diseases occurring due to the α_1D_-AR [23]. Antagonists of the α_1_-AR subtypes are important as treatments of many kinds of diseases. However, antagonists have many kinds of effects [49]. Antagonists of the α_1A_-AR inhibit phenylephrine-induced salivary secretion, resulting in xerostomia or dry mouth [24]. In the present study, we showed the inhibitory mechanism in relation to loss of AQP5 trafficking. 

The maximal amount of AQP5 protein in the APM of rat parotid glands stimulated by muscarinic receptor and α_1_-AR agonists was 3- [11,37] and 1.8-fold higher than unstimulated condition, respectively. Ten percent of patients who received silodosin orally (4 mg twice daily) felt thirsty, whereas in placebo group only 4.5% of patients had the same symptom [19]. At therapeutic dose (7.5 mg once daily) of darifenecin (muscarinic antagonist), 20% of patients with LUTS suffered from dry mouth, while 4.5% of patients in placebo group experienced this adverse effect [50]. In the treatment of LUTS, muscarinic antagonists contribute to the higher incidence of dry mouth compared with α_1A_-adrenergic antagonists, which may be correlated with proportionally higher stimulatory effect of mAChR agonists on AQP5 trafficking in parotid gland. 

The α_1_-AR couples to different signaling pathways [25,26]. The α_1A_-AR couples to phosphatidylinositol turnover such as M_1_ and M_3_-mAChR signaling and the subsequent rise in [Ca^2+^]i [45,51], while the α_1B_-AR activates MAPK/ERK1/2 [26]. In the Ca^2+^-mediated intracellular signaling pathway mechanisms, an increase in [Ca^2+^]i plays an essential role in the stimulation of Ca^2+^/calmodulin (CaM)-dependent proteins, e.g., myosin light chain kinase, CaM kinases, and NO synthase [52,53]. It is generally recognized that NO activates GC, producing cGMP, which then activates PKG [1,54]. In this study, to examine the possible role of cGMP/PKG signal transduction on AQP5 levels in the APM of phenylephrine-stimulated parotid glands, the tissue was incubated with ODQ or KT5823, inhibitors of NO-stimulated guanylyl cyclase and PKG, respectively. The results revealed that phenylephrine-induced trafficking of AQP5 to the APM was attenuated by the use of specific inhibitors, suggesting the involvement of cGMP and PKG in this mechanism. 

Recently, aquaporin levels have been shown to be dynamically regulated by hypo- and hypertonicity-induced [Ca^2+^]i increases in different cell types [28,29,30,31,55]. In the present study, we investigated the responsiveness of AQP5 trafficking to phenylephrine under isotonic and hypotonic conditions. Under isotonic conditions, La^3+^, but not RR, inhibited phenylephrine-induced trafficking of AQP5, suggesting that SOCE may regulate α_1_-AR-induced trafficking of AQP5 to the APM in parotid glands. RR and La^3+^ inhibited hypotonic-induced trafficking of AQP5 to the APM, suggesting that TRPV activation and extracellular influx of Ca^2+^ may be required in this induction. Several studies describe inhibitory effect of RR and La^3+^ on the hypotonicity-induced increase in [Ca^2+^]i via activation of TRPV [41]. In parotid glands, hypotonicity induced trafficking of AQP5 to the APM and LPM within minutes in consistency with data described previously [28]. In lung epithelial cells, hypotonicity reduced the AQP5 abundance mediated by TRPV4 channel over a period of hours, supposing that hypotonicity induced the trafficking of AQP5 to lysosomes [29]. The basis of this difference is not clearly known, however the duration time of incubation in hypotonic condition might explain the apparent discrepancy since long term exposure to osmotic stress has deleterious consequences on protein degradation [56]. Moreover, NO donors [37] and NO induce trafficking of AQP5 to the APM in rat parotid glands, but NO decreases cell surface expression of AQP5 in lung epithelial cells [57]. Differential signal transductions may affect trafficking of AQP5 in parotid glands and lung epithelial cells. Furthermore, our studies showed that under hypotonic conditions, phenylephrine induced a 2.4-fold higher trafficking of AQP5 to the APM than the control, suggesting that both SOCE and TRPV may be involved in hypotonic condition. Taken together, SOCE and TRPV contributes to the induction of AQP5 trafficking to APM probably via distinct mechanisms, however, connected by the indispensable role of [Ca^2+^]i.

In the present study, we showed that phenylephrine-induced trafficking of AQP5 required activation of PKG and enhancement of [Ca^2+^]i in parotid glands. In parotid glands, activation of α_1_-ARs [51] and mAChRs [58], but not β-ARs [59,60], was reported to elevate [Ca^2+^]i levels. [Ca^2+^]i is referred to as store-operated Ca^2+^ pool that is activated by the release of endoplasmic reticulum Ca^2+^, which initiate SOCE [61]. Our previous studies showed that intravenous injection of A-23187 (Ca^2+^ ionophore which induces an increase in the [Ca^2+^]i), but not the incubation of the parotid tissues with A-23187, induces trafficking of AQP5 to the APM [12], indicating that the enhancement of [Ca^2+^]i is necessary but not sufficient for the translocation of AQP5 to APM. As described previously by us [4,10], and others [62], lipid rafts were also required for AQP5 trafficking to the APM in parotid tissues. Upon activation of α_1A_-AR, anchor proteins may mediate lipid raft-dependent trafficking of AQP5 to the APM and LPM via the enhancement of [Ca^2+^]i in parotid glands. Although Rab 4 protein is reported to be co-localized with AQP5 in parotid glands [63], Ca^2+^-dependent phospholipid-binding proteins are also assumable to work as anchor protein for membrane-recruitment of AQP5. 

Together our present data and previous studies [8,9,11,12,37] clearly indicate the involvement of both mAChRs and α_1_-ARs in AQP5 trafficking to APM and LPM and its contribution to saliva secretion in rat parotid tissue. Indeed, parasympathetic nerves usually evoke most of the salivary fluid secretion, while sympathetic nerves have less of a fluid evoking role. However, dual stimulation is expecting to have better synergistic effect on secretion of large volume of fluid secretion across the plasma membrane [1]. Therefore, the effects of impulses from sympathetic and parasympathetic nerves on salivary secretion should be no longer dichotomized but rather considered as an integrated signaling platform necessary for the proper function of salivary glands.

## 4. Materials and Methods

### 4.1. Materials

Phenylephrine hydrochloride and L765314 were obtained from Sigma Aldrich (Munich, Germany). Phentolamine hydrochloride was purchased from Ciba-Geigy Japan (Tokyo, Japan). Silodosin and Urief 4 mg capsule were obtained from Kissei Pharmaceutical Co., Ltd. (Azumino City, Japan) and Daiichi Sankyo Company (Tokyo, Japan), respectively. 8-[2-[4-(2-Methoxyphenyl)-1-piperazinyl]ethyl]-8-azaspiro[4.5]decane-7,9-dione dihydrochloride (BMY7378) was purchased from Funakoshi Co. (Tokyo, Japan). 1H-[1,2,4]Oxadiazolo[4,3-a]quinoxalin-1-one (ODQ) and KT5823 were from Calbiochem-Behring Co. (La Jolla, CA, USA). Ruthenium red and lanthanum chloride heptahydrate were purchased from TCI (Tokyo, Japan) and Nacalai Tesque (Kyoto, Japan), respectively.

### 4.2. Animals

Eight-week-old male Wistar rats from SLC, Inc. (Shizuoka, Japan) were provided with standard laboratory chow (MF; Oriental Yeast, Tokyo, Japan) and water ad libitum, and were maintained in a temperature-controlled environment (22 ± 2 °C) with a 12-h dark/light cycle. All procedures were performed in accordance with the guidelines established by the Animal Care Committee of Tokushima University (Approval NO.:14120) (Tokushima, Japan).

### 4.3. Immunohistochemistry

Phenylephrine hydrochloride (0.25 mg/kg) [64] was intravenously injected into the rat tail vein. At 0, 3, 6, 10 min after the injection, parotid glands were quickly removed, embedded in Jung tissue freezing medium (Leica, Heidelberg, Germany), and rapidly frozen with liquid nitrogen. In another group of animals, phenylephrine (0.25 mg/kg) was injected 60 min after intraperitoneal injection of phentolamine hydrochloride (10 mg/kg) [65]. In the last group of rats, phenylephrine (0.25 mg/kg) was injected after oral administration of silodosin at a daily dose of 1 mg/kg for 1 week [20]. Frozen sections (7-µm thick) were cut, mounted on poly-l-lysine-coated glass slides, and immediately fixed for 30 min with prechilled (−20 °C) ethanol. After washing in phosphate buffered saline (PBS, pH 7.5), sections were blocked by serum-free protein block (Dako, North America Inc., Carpinteria, CA, USA) for 20 min, and stained as follows. Sections were incubated overnight at 4 °C with primary antibodies: rabbit anti-AQP5 antibody (1:1000 dilution) generated in response to a synthetic peptide (KGTYEPEEDWEDHREERKKTI) corresponding to the C-terminal amino acid sequence of AQP5 [8], rabbit anti-ganglioside GM1 antibody (1:1000 dilution; Calbiochem-Novabiochem Co., Darmstadt, Germany) and mouse anti-*E*-cadherin antibody (1:50 dilution; BD Bioscience, San Diego, CA, USA). The labeling was visualized by incubation for 1 h with Alexa Fluor 488 donkey anti-rabbit and/or Alexa Fluor 568 goat anti-mouse secondary antibody (1:1000 dilution; Alexa 488/568; Molecular Probes Europe, Leiden, The Netherlands). To stain cell nuclei, sections were incubated with 0.5 µg/mL RNase A and then with 2 µg/mL of PI for 1 h at 37 °C. Fluorescence images were captured using a confocal laser scanning microscope (Leica TCS NT, Wetzlar, Germany).

### 4.4. Preparation of Rat Parotid Glands

Rats were anesthetized with chloroform and sacrificed by a blow to the head. The parotid glands (300 mg wet weight) were quickly removed and placed in ice-cold Krebs-Ringer-Tris (KRT) buffer containing 120 mM NaCl, 4.8 mM KCl, 1.2 mM KH_2_PO_4_, 1.2 mM MgSO_4_, 1.0 mM CaCl_2_, 16 mM Tris-HCl, pH 7.4, and 5 mM glucose, which was aerated with O_2_ gas before the experiment. The 0.4-mm thick slices were prepared from the parotid glands using a McIlwain Tissue Chopper (Mickle Laboratory Engineering, Surrey, UK) followed by equilibrated with KRT buffer for 20 min at 37 °C with shaking, as described previously [11]. 

#### 4.4.1. Incubation of Rat Parotid Gland Slices

The slices prepared from parotid tissue were further incubated for 10 min at 37 °C in KRT solution with or without phenylephrine and/or phentolamine, silodosin, L765314, BMY7378, ODQ, KT5823, RR, and La^3+^. Parotid tissues were also incubated in iso-, hypo-, and hypertonic solutions (10 min). KRT solution was made hypertonic or hypotonic by the addition of higher tonicity solution and by dilution with water, respectively. The following KRB solutions were used in the osmolality experiment: isotonic (264 mOsm/kg, 120 mM NaCl), hypertonic KRT-NaCl (491 mOsm/kg, 240 mM NaCl and 700 mOsm/kg, 360 mM NaCl), hypotonic KRT-NaCl (132 mOsm/kg, 60 mM NaCl and 87 mOsm/kg, 40 mM NaCl). The tissue in the hypotonic solution was additionally incubated in the presence of ruthenium red or phenylephrine.

#### 4.4.2. Preparation of APM from Parotid Gland Slices

The APM fraction was prepared from rat parotid glands according to the modified method of Longbottom and Van Heyningen [66]. In brief, after incubation, parotid gland slices were homogenized using a glass homogenizer and a Teflon pestle in 20 volume of 5 mM HEPES buffer, pH 7.5, containing 50 mM mannitol and 0.25 mM MgCl_2_, followed by filtration of the homogenate through 150 mesh-nylon bolting cloth. The filtrate was processed to differential centrifugation steps, and the pellet collected after centrifugation at 35,000× *g* for 30 min was suspended in the buffer described above. After the addition of 1 M MgCl_2_ to give a final concentration of 10 mM, the suspension was incubated for 30 min on ice with stirring followed by centrifugation at 3000× *g* for 15 min. The resultant precipitate was marked as BLM and the supernatant was again centrifuged at 35,000× *g* for 30 min for precipitation of APM. To assess the purity of APM, the isolated membrane fractions were assayed for the activities of γ-glutamyltranspeptidase (γ-GT) as a marker of the APM and K^+^-stimulated *p*-nitrophenyl-phosphatase as a marker of BLM according to methods of Meister et al. [67] and Alves et al. [68], respectively. The activity was expressed as micromoles of p-nitroaniline or p-nitrophenol released per minute (units) per mg of protein.

### 4.5. Immunoblot Analysis

The APM fraction was subjected to SDS-polyacrylamide gel electrophoresis (PAGE) on a 12.5% gel. The separated proteins were transferred to a nitrocellulose membrane (Hybond ECL; Amersham Biosciences, Little Chalfont, Buckinghamshire, UK) using a Trans-Blot apparatus (Bio-Rad, Hercules, CA, USA). Blots were stained with Ponceau S (Nacalai Tesque, Kyoto, Japan) dye to confirm equal protein content in all lanes. Membranes were soaked in PBS to remove the Ponceau S and blocked with 0.1% Tween 20 in Tris-buffered saline, pH 7.4, overnight. Blots were then probed with antibody against AQP5 (1:1500). Immune complexes were detected with horseradish peroxidase-conjugated donkey anti-rabbit IgG, whole Ab (1:3000, GE Healthcare, Piscataway, NJ, USA) and ECL reagents (Amersham Pharmacia Biotech, Little Chalfont, UK). Chemiluminescence was measured using a Chemi Doc apparatus (Bio-Rad, Hercules, CA, USA), analyzed using Quantity One software (Bio-Rad, Hercules, CA, USA), and normalized by Ponceau S staining [34,35].

### 4.6. Osmolality

The osmolality of all solutions employed was measured using a vapor pressure osmometer (Vescor, model 5520; Logan, UT, USA).

### 4.7. Statistical Analysis

Data are presented as mean ± standard error (SE), and were analyzed for statistical significance using Student’s *t*-test or analysis of variance at all-time points. *p* Values < 0.05 were considered statistically significant.

## 5. Conclusions

Rapid phenylephrine-induced AQP5 translocation to APM and LPM of rat parotid tissue occurred via activation of α_1A_-AR subtype, but not α_1__B_- and α_1D_-AR subtypes. The process was mediated by the Ca^2+^/cGMP/PKG signaling pathway, which was associated with SOCE. Under non-stimulated conditions, hypotonicity induced the trafficking of AQP5 to the APM. The extent of responsiveness of AQP5 trafficking to an α_1_-AR agonist under hypotonic condition was the same as that under isotonic condition.

## Supplementary Materials

Supplementary materials can be found at http://www.mdpi.com/1422-0067/17/7/1022/s1.

## Abbreviations

ACh	Acetylcholine
APM	Apical plasma membrane
AQP	Aquaporin
AR	Adrenergic receptor
BLM	Basolateral plasma membrane
BMY7378	8-[2-[4-(2-methoxyphenyl)-1-piperazinyl]ethyl]-8-azaspiro[4.5]decane-7,9-dione dihydrochloride
CaM	Calcium/calmodulin-dependent kinase
cGC	Soluble guanylate cyclase
cGMP	Cyclic guanosine monophosphate
γ-GT	γ-glutamyl transpeptidase
GM	Monosialotetrahexosylganglioside
HEK293	Human Embryonic Kidney 293 cells
HEPESr	4-(2-hydroxyethyl)-1-piperazineethanesulfonic acid
K^+^-NPPase	K^+^-stimulated *p*-nitrophenyl phosphatase
KRT buffer	Krebs-Ringer-Tris buffer
KT5823	(9*S*,10*R*,12*R*)-2,3,9,10,11,12-Hexahydro-10-methoxy-2,9-dimethyl-1-oxo-9,12-epoxy-1H-diindolo[1,2,3-fg: 3′,2′,1′-kl]pyrrolo[3,4-i][1,6]benzodiazocine-10-carboxylic acid, methyl ester
L765314	[(2S)-4-(4-amino-6,7-dimethoxy-2-quinazolinyl)-2-[[(1,1-dimethylethyl)amino]carbonyl]-1-piperazinecarboxylic acid]
LPM	Lateral plasma membrane
LUTS	Lower urinary tract symptoms
mAChR	Muscarinic acetylcholine receptor
NOS	Nitric-oxide synthase
ODQ	1H-[1,2,4]oxadiazolo[4,3-a]quinoxalin-1-one
Orai	Calcium release-activated calcium channel protein
PBS	Phosphate buffered saline
PE	Phenylephrine
PK	Protein kinase
RR	Ruthenium red
SDS-PAGE	Sodium dodecyl sulfate polyacrylamide gel electrophoresis
SOCE	Store operated calcium entry
STIM	Stromal Interaction Molecule
TRPC	Transient receptor potential channel
TRPV	Transient receptor potential vanilloid
