# Supplementary material for: Mechanisms Underlying Activation of α1-Adrenergic Receptor-Induced Trafficking of AQP5 in Rat Parotid Acinar Cells under Isotonic or Hypotonic Conditions
Source: Int J Mol Sci. 2016 Jun 28;17(7):1022. doi: 10.3390/ijms17071022 (PMC4964398; doi:10.3390/ijms17071022)
Supplement: Supplementary file 1 [file ijms-17-01022-s001.pdf]

Supplementary Materials: Mechanisms Underlying Activation of α_1-Adrenergic Receptor-Induced Trafficking of AQP5 in Rat Parotid Acinar Cells under Isotonic or Hypotonic Conditions

Aneta M. Bragiel, Di Wang, Tomasz D. Pieczonka, Masayuki Shono and Yasuko Ishikawa

Table S1. Activities of γ-glutamyl transpeptidase and K⁺-activated *p*-nitrophosphatase in subfractions.

Fractions	γ-Glutamyl Transpeptidase (mU/mg Protein)	K^+-Activated *p*-Nitrophosphatase (mU/mg Protein)
Homogenate	3.53 ± 0.31	34.47 ± 1.62
APM	24.23 ± 1.0	19.56 ± 1.81
BLM	4.00 ± 0.06	109.09 ± 4.11
Cytoplasm	2.11 ± 0.04	15.15 ± 0.61

APM: apical plasma memnrane; BLM: basolateral plasma membrane; *n* = 8.

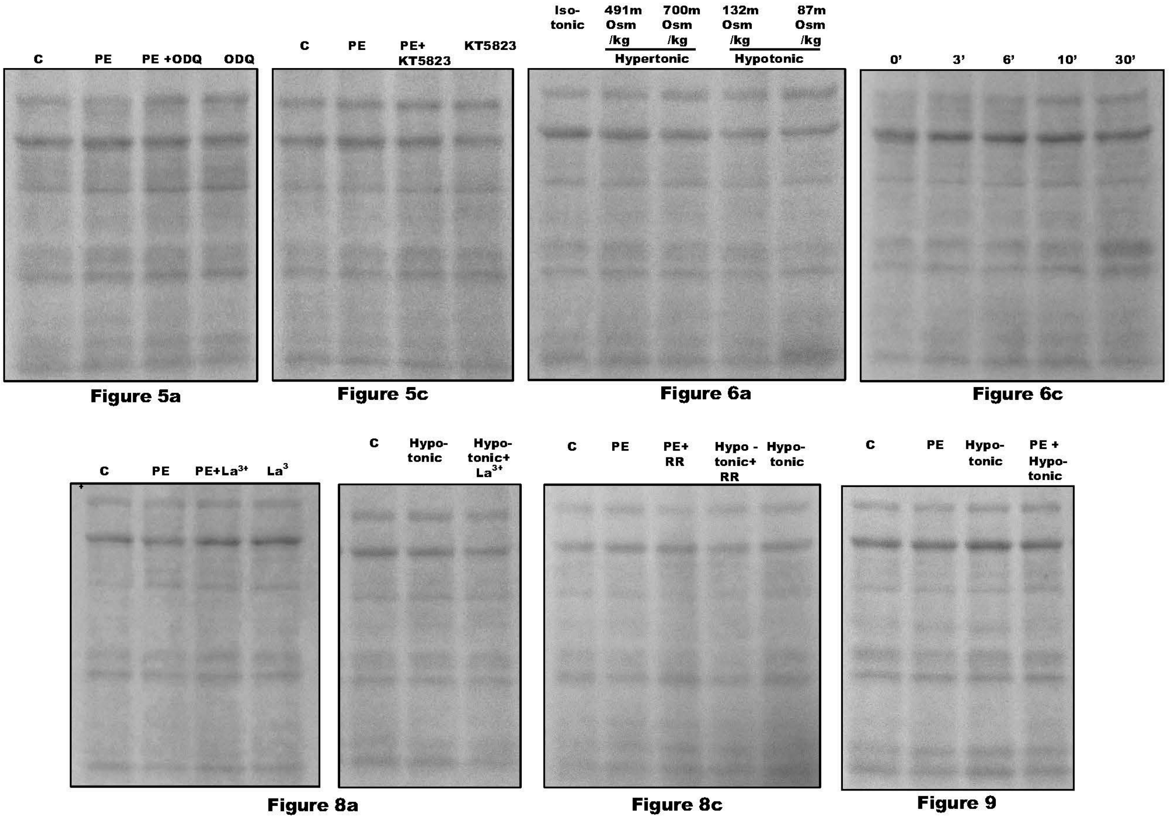

Figure S1. Protein loading determination by Ponceau S staining of nitrocellulose membranes of APM (apical plasma memnrane) samples. The 5 μg of APM fraction protein was loaded on SDS-PAGE and processed by immunoblot analysis with anti-AQP5 antibody. Nitrocellulose membrane was stained with Ponceau S. Each number of figure S1 accords with the number of immunoblot analysis in the text.
